# Association of Chain Restaurant Advertising Spending With Obesity in US Adults

**DOI:** 10.1001/jamanetworkopen.2020.19519

**Published:** 2020-10-07

**Authors:** Sara N. Bleich, Mark J. Soto, Jesse C. Jones-Smith, Julia A. Wolfson, Marian P. Jarlenski, Caroline G. Dunn, Johannah M. Frelier, Bradley J. Herring

**Affiliations:** 1 Department of Health Policy and Management, Harvard T.H. Chan School of Public Health, Boston, Massachusetts; 2 Department of Health Services, University of Washington School of Public Health, Seattle; 3 Department of Health Management and Policy, Department of Nutritional Sciences, University of Michigan School of Public Health, Ann Arbor; 4 Department of Health Policy and Management, University of Pittsburgh Graduate School of Public Health, Pittsburgh, Pennsylvania; 5 Department of Health Policy and Management, Johns Hopkins Bloomberg School of Public Health, Baltimore, Maryland

## Abstract

**Importance:**

Restaurants spend billions of dollars on marketing. However, little is known about the association between restaurant marketing and obesity risk in adults.

**Objective:**

To examine associations between changes in per capita county-level restaurant advertising spending over time and changes in objectively measured body mass index (BMI) for adult patients.

**Design, Setting, and Participants:**

This cohort study used regression models with county fixed effects to examine associations between changes in per capita county-level (370 counties across 44 states) restaurant advertising spending over time with changes in objectively measured body mass index (BMI) for US adult patients from 2013 to 2016. Different media types and restaurant types were analyzed together and separately. The cohort was derived from deidentified patient data obtained from athenahealth. The final analytic sample included 5 987 213 patients, and the analysis was conducted from March 2018 to November 2019.

**Exposure:**

Per capita county-level chain restaurant advertising spending.

**Main Outcomes and Measures:**

Individual-level mean BMI during the quarter.

**Results:**

The included individuals were generally older (37.1% older than 60 years), female (56.8%), and commercially insured (53.5%). For the full population of 29 285 920 person-quarters, there was no association between changes in all restaurant advertising per capita (all media types, all restaurants) and changes in BMI. However, restaurant advertising spending was positively associated with weight gain for patients in low-income counties but not in high-income counties. A $1 increase in quarterly advertising per capita across all media and restaurant types was associated with a 0.053-unit increase in BMI (95% CI, 0.001-0.102) for patients in low-income counties, corresponding to a 0.12% decrease in BMI at the 10th percentile of changes in county advertising spending vs a 0.12% increase in BMI at the 90th percentile.

**Conclusions and Relevance:**

The results of this study suggest that restaurant advertising is associated with modest weight gain among adult patients in low-income counties. To date, there has been no public policy action or private sector action to limit adult exposure to unhealthy restaurant advertising. Efforts to decrease restaurant advertising in low-income communities should be intensified and rigorously evaluated to understand their potential for increasing health equity.

## Key Points

**Question:**

Does an association exist between restaurant marketing and obesity risk in US adults?

**Findings:**

In this longitudinal cohort study of 29 285 920 person-quarters, a $1 increase in quarterly advertising per capita across all media and restaurant types was not associated with changes in body mass index overall but was associated with a 0.053-unit increase in body mass index for patients in low-income counties across 44 states.

**Meaning:**

These study results suggest that restaurant advertising is associated with modest weight gain among US adults in low-income counties.

## Teaser

This cohort study assesses whether changes in per capita county-level restaurant advertising spending from 2013 to 2016 are associated with changes in objectively measured body mass index in nearly 6 million US adults across 370 counties.

## Introduction

Food and beverage marketing, which primarily promotes unhealthy foods, has been identified as a suspected risk factor for obesity.^1,2,3^ A key mechanism by which food and beverage marketing increases obesity risk is by changing consumer preferences, which are highly malleable.^3^ Targeted food marketing identifies consumers who share common characteristics and positions products to appeal to these consumers.^4^ Research shows that marketing for unhealthy food and beverage items is more often targeted toward racial/ethnic minority and low-income populations.^5,6^ This is particularly concerning because it may exacerbate long-standing obesity disparities, particularly those based on race/ethnicity and income.^7,8^

Restaurants are an important component of food and beverage marketing. In 2018, the top 10 revenue-generating US restaurants represented 23.3% of the total market and spent $6.2 billion on advertising.^9^ Adults viewed a mean of 2800 restaurant advertisements in 2015,^10^ most of which appeared on television (TV).^9^ Spending on food away from home accounts for more than half of American’s food budgets, and 72% of money spent on food outside the home is spent at restaurants.^11^ Americans spent more than $863 billion at restaurants in 2019,^12^ and one-third of Americans will eat at a fast food restaurant on a typical day.^13^ This adversely affects health because consumption of restaurant food, particularly fast food, is linked to higher intake of calories, fat, sodium, and cholesterol.^14^

To date, research on the association between restaurant advertising and obesity risk has largely focused on children;^1,2,15,16,17,18,19,20,21,22,23^ very little is known about this association among adults. Prior research is also limited by making cross-sectional comparisons,^20,22,24,25,26^ which generally measure preference, purchases, or intake and do not allow for the observation of health-related outcome changes. To help fill these gaps and expand on existing evidence, we created a county-level measure of restaurant advertising per capita, scaled to account for restaurant density, and examined associations between changes in this measure over time and changes in body mass index (BMI, calculated as weight in kilograms divided by height in meters squared) for a longitudinal cohort of adult patients. We also examined differences across counties based on income and examined different advertising exposure measures based on restaurant type (fast food, fast causal, and full service) and media type (eg, TV, radio, and internet).

## Methods

We examined individual-level BMI from 2013 to 2016. Our primary independent variable was a county-level measure of quarterly per capita spending on restaurant advertising; our models included county fixed effects so that our analyses examined the association of changes in restaurant spending over time with changes in BMI. We combined data from several sources, which are described below (additional details are in eTable 1 in the Supplement). This study followed the Strengthening the Reporting of Observational Studies in Epidemiology (STROBE) reporting guideline for cohort studies. This study was determined to be non–human subjects research by the Harvard University Institutional Review Board.

### Sample

We obtained deidentified patient data from athenahealth, a health care services and technology company with more than 85 000 primary care physicians primarily situated in outpatient settings. Data were extracted from the athenahealth ambulatory electronic health record, which tracks patients with a unique, anonymized patient identifier. All of the medical encounters were primary care visits.

Patient records were eligible to be included in our extract if the patient was 20 years of age or older at the start of the study period in 2013 and visited an athenahealth primary care physician at least once during the 2013 to 2016 study period in which their BMI was recorded (7 522 351 patients) (eFigure in the Supplement). Patients were assigned to the county where they received health care because the county of patient residence was not included in these data. Patients were excluded if they received care in more than 1 county during the study period (n = 88 994) or the county identifier was suppressed for privacy concerns (n = 9985). Patients were also excluded if their assigned county was not present in the data for all 16 quarters between 2013 and 2016 (n = 1 435 954); these counties were excluded to yield a consistent sample of counties during all 16 quarters. Patients were also excluded if they received care in a county with less than 10 patients in all 16 quarters (n = 158) or if they were missing individual-level demographic characteristics (eg, sex) (n = 47). Patients who were pregnant (based on diagnosis codes) were excluded prior to the research team receiving the data. The final analytic sample included 5 987 213 patients receiving health care in 370 counties.

### Measures

The outcome of interest was individual-level mean BMI during the quarter. Height and weight data used to calculate BMI were measured during clinic visits and recorded in the electronic health record. If there were multiple BMI observations within a given quarter, a mean BMI was computed. For quarters without a measured BMI, a linear interpolation between recorded values was computed; 37.8% of quarterly BMI measurements in the sample were imputed. We did not include extrapolated BMI values before the earliest record or after the latest record.

The independent variable of interest was a county-level measure of quarterly restaurant advertising spending per capita. Data used to construct this variable were obtained from several sources (additional details are in eTable 1 in the Supplement). We used AggData^27^ to identify the zip code locations for a majority of the top 100 grossing US chain restaurants from 2013 to 2016 and obtained quarterly advertising spending data during this same period from Kantar Media,^28^ which tracks advertisements for more than 7500 websites, 4000 radio stations, 1600 magazines, 1000 TV stations, and 230 newspapers based on rate cards provided by publishers, TV and radio networks, and advertising agencies or media cost forecasting data. The county population was obtained from the American Community Survey conducted by the US Census Bureau.

Kantar Media provides quarterly data for both local advertising spending (at the level of designated market areas [DMAs], which generally encompass several counties) and national advertising spending in dollars. We first disaggregated these quarterly advertising dollars for each restaurant chain to individual counties based on the number of locations for each restaurant chain in a given county using AggData. Specifically, for each restaurant chain, we calculated the proportion of restaurant locations in a given county by dividing the total number of locations in a county by the total number of locations in a DMA. Then, we multiplied the proportion of all locations within a county in a given year by the total advertising expenditures for that restaurant chain per quarter. We then summed those disaggregated chain-specific spending amounts across all restaurant chains with locations in that county to determine total restaurant advertising in a county per quarter. We then divided the measure of total spending by the county population to determine per capita quarterly spending. Our subsequent empirical analyses focused on the changes over time in quarterly advertising per capita within a given county. More details, along with an example of this calculation, are included in the eAppendix and eTable 2 in the Supplement. We also produced additional secondary measures of advertising spending per capita by distinguishing restaurant types (ie, fast food, fast casual, and full service) based on definitions previously established in public health research,^29^ and by distinguishing media type (eg, TV, radio, and internet) based on categories from Kantar Media.

Patient-level demographic characteristics in the athenahealth data included age, sex, race/ethnicity, and insurance type. Patient age was assigned in 2013 and consolidated by athenahealth into 10-year age intervals for those between 20 and 59 years of age and 1 category for those older than 60 years. Patients self-identified their sex as either male or female. Primary race/ethnicity was coded as a mutually exclusive categorical variable, with patients self-identifying as either White, Black/African American, Hispanic/Latino/Spanish, or Asian. Black and Hispanic patients were grouped together into a single category owing to a small sample of Hispanic respondents (n = 6735). Insurance type was either commercial, Medicaid, Medicare, self-pay, or other.

County-level socioeconomic covariates included annual measures of median household income, educational attainment, and unemployment. Educational attainment was measured as the percentage of county residents with at least 4 years of college; unemployment was calculated as the percentage of unemployed individuals older than 16 years as a percentage of the county’s workforce.

### Statistical Analysis

Ordinary least-squares regression models were analyzed from March 2018 to November 2019 using Stata, version 15 (StataCorp). A 2-sided *P* < .05 was considered statistically significant. The unit of observation in these models was the person-quarter, the primary outcome was an individual’s BMI, and the primary predictor was a county-level per capita measure of restaurant advertising; robust standard errors accounted for clustering within counties. Models included individual-level factors (age, sex, race/ethnicity, and insurance), time-varying county-level factors (median income, educational level [ie, percentage with college degree], and percentage of unemployment), and county fixed effects to account for time-invariant differences between counties. In addition to examining the impact of restaurant advertising across the full sample of patients, we stratified patients into low-income or high-income subsamples of counties based on the county being below or above the median value in median household income in 2013.

We also examined models in which the primary estimator, county-level per capita restaurant advertising, was constructed for specific subsets of restaurants and media types (both separately and interacted with each other). We primarily identified restaurants as either fast food or fast casual or full service based on definitions used in prior research,^29,30,31^ and we primarily identified media types as either TV only or all other media. We also performed additional sensitivity analyses distinguishing all 3 restaurant types and all 6 media types separately. We based our categorization of these primary subsets for both restaurant type and media type on the relative magnitudes of spending observed in the Kantar Media data, which were consistent with previous research showing that a disproportionate amount of money is spent on advertising for fast food restaurants (compared with fast casual or full service) and on TV (compared with all other media types).^6^ Finally, we conducted a sensitivity analysis in which we reestimated the main analyses excluding 125 843 patients assigned to 1 of the 39 rural counties to investigate whether inherent differences between urban and rural settings potentially skewed the main results. Although a measure of restaurant advertising per capita may not be comparable between urban/suburban vs rural settings cross-sectionally, our primary analysis of changes in advertising per capita over time should not be problematic; that said, we still conducted these sensitivity analyses excluding rural counties.

To contextualize the interpretation of our results, we present estimated changes in BMI associated with 3 points in the distribution of changes in restaurant advertising over time—specifically the change in restaurant advertising at the 10th percentile, the mean, and the 90th percentile of per capita advertising spending. This analysis combined the results from the regression models with descriptive statistics for our sample for the outcome (ie, mean BMI) and primary independent variables of interest (ie, marketing advertising per capita). We also translated those estimated changes in BMI to the change in kilograms from assuming the average height of 1.62 m (5 feet and 3.6 inches) and weight of 72.8 kg (170.5 pounds) for women in the US.^32^

## Results

**Figure 1.**
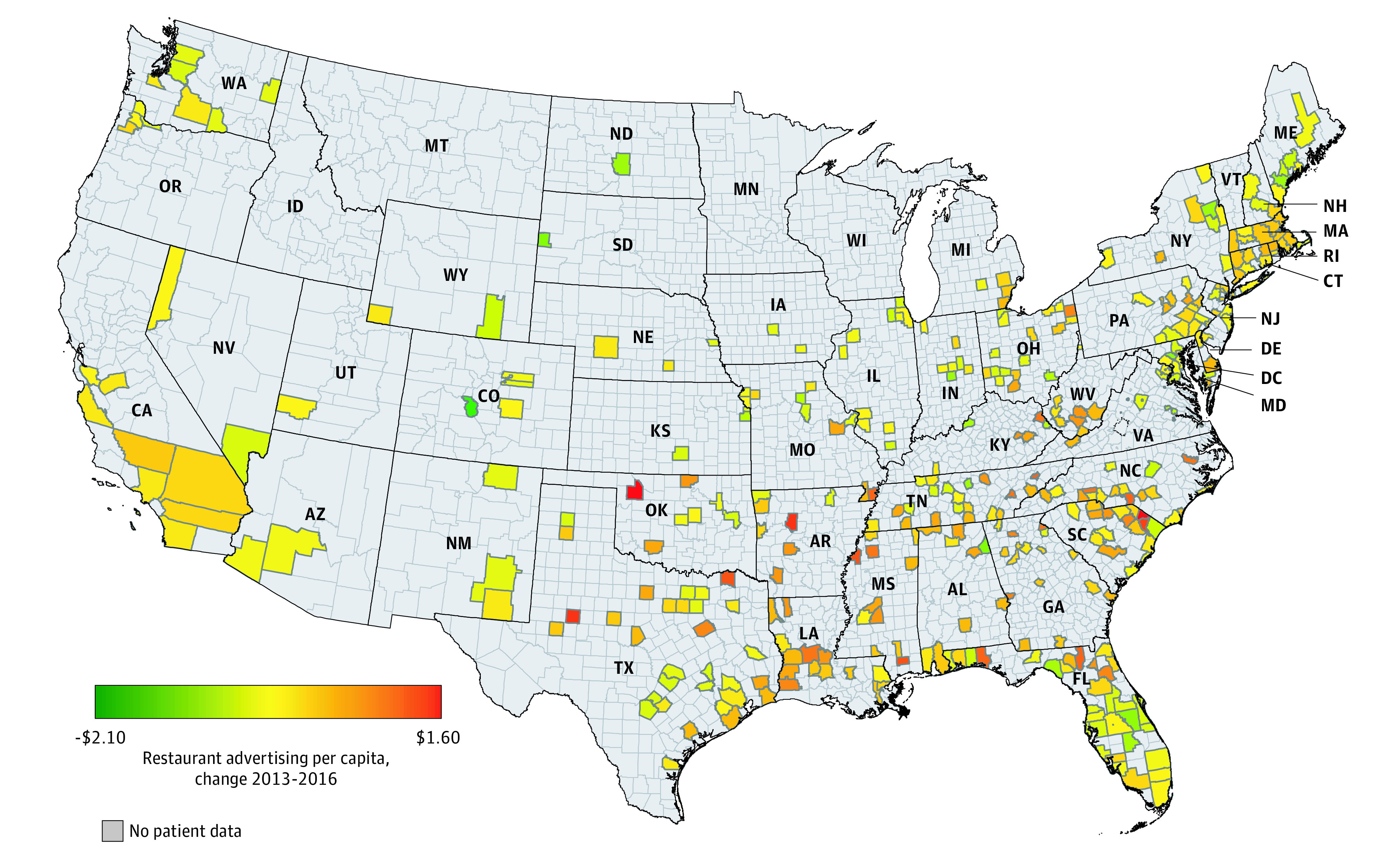
Distribution of Changes in Quarterly Restaurant Advertising Dollars Per Capita, 2013-2016 Variation across counties in the changes in advertising for all media and all restaurant types in the 370 counties included in our final sample of athenahealth patients is illustrated for 2013 vs 2016. The source of identification for the regression models presented in Table 2 is quarter-to-quarter changes in advertising during this period. Because advertising dollars are not consistently spent across all quarters in 1 year, the county-level estimates computed a mean of all quarters for 2013 and 2016 and calculated a difference in mean yearly spending per capita, which ranged from a low of −$2.10 (darkest green) to a high of $1.60 (darkest red). Gray areas represent counties without patient electronic health records. The changes from 2013 to 2016 presented here are not necessarily monotonic changes during the 16 quarters within the 4-year period used for the regression analysis.

Patient and county characteristics across the 2013 to 2016 study period are presented in Table 1. During the 16-quarter study period, the mean BMI (SD) was 29.8 (6.9), which is classified as overweight. The sample was generally older (37.1% older than 60 years), female (56.8%), and commercially insured (53.5%). Patients received health care in counties where a mean (SD) 26.5% (11.4%) of the population had attended at least 4 years of college, with a mean (SD) 7.4% (2.1%) unemployment, and with a median (SD) household income of $50 450 ($14 440). Overall restaurant advertising per capita was a mean (SD) $4.72 ($1.61) per quarter, and spending was higher in low-income counties compared with high-income counties (mean [SD] spending, $4.95 [$1.81] vs $4.50 [$1.36]). The majority of this money was spent on fast food advertising (mean [SD] spending, $3.52 [$1.16] per person per quarter) and on TV advertising (mean [SD] spending, $4.24 [$1.52] per person per quarter). Figure 1 shows that considerable variation existed across counties in the changes in mean quarterly per capita restaurant advertising spending between 2013 and 2016 for the counties included in our final sample of patients in the athenahealth data.

**Table 1.** Individual- and County-Level Characteristics, 2013-2016^a^

Characteristic	% (SD) of included patients^b^		
	Full sample	Low income	High income
Individual patients			
No.	29 285 920	10 015 358	19 270 562
BMI, mean (SD)	29.8 (6.9)	30.2 (7.1)	29.6 (6.8)
Age group, %			
20-29 y	9.7	8.2	10.5
30-39 y	12.9	11.1	13.8
40-49 y	18.1	16.6	18.9
50-59 y	22.2	22.1	22.2
≥60 y	37.1	42.0	34.5
Sex			
Female	56.8	57.2	56.6
Male	43.2	42.8	43.4
Insurance type			
Commercial	53.5	45.0	58.0
Medicare	34.8	42.1	31.0
Medicaid	7.1	7.1	7.1
Self-pay	3.8	5.0	3.2
Other	0.8	0.9	0.8
County-level restaurant advertising exposure, Kantar Media dollars spent per person-quarter			
No.	5920	2960	2960
Overall	4.72 (1.61)	4.95 (1.81)	4.50 (1.36)
By restaurant type			
Fast food	3.52 (1.16)	3.77 (1.28)	3.26 (0.97)
Fast casual	0.22 (0.19)	0.17 (0.19)	0.26 (0.18)
Full service	0.99 (0.64)	1.00 (0.74)	0.98 (0.51)
By media type			
Television	4.24 (1.52)	4.54 (1.66)	3.94 (1.29)
Internet	0.08 (0.04)	0.08 (0.04)	0.08 (0.04)
Business to business	0.001 (0.001)	0.001 (0.001)	0.001 (0.0005)
Outdoor	0.15 (0.11)	0.14 (0.12)	0.15 (0.10)
Print	0.09 (0.05)	0.08 (0.05)	0.10 (0.05)
Radio	0.17 (0.15)	0.11 (0.12)	0.23 (0.16)
County level			
No.	1480	740	740
Median income, $	50 450 (14 440)	39 770 (4660)	61 140 (12 910)
≥4 Years of college	26.5 (11.4)	19.3 (7.1)	33.7 (10.4)
Unemployment rate	7.4 (2.1)	8.2 (2.4)	6.7 (1.5)

Abbreviation: BMI, body mass index calculated as weight in kilograms divided by height in meters squared.

^a^ Analysis of quarterly per capita restaurant advertising between 2013 and 2016 for the 370 counties included in the final sample of patients in the athenahealth database.

^b^ County income level determined based on the median value of the median household income in 2013 of $46 832.

The results of regression models analyzing the association of changes in per capita restaurant advertising with changes in BMI are provided in Table 2. The first column gives the results for the full sample, and the next 2 columns provide the results stratified by low-income and high-income subsamples. The first row indicates the results for the advertising measure including all restaurant types and media types, whereas the subsequent rows give results from the advertising measures distinguished across restaurant types, across media types, and across both restaurant types and media types jointly. For the full population, there was no association between changes in all restaurant advertising per capita (all media types, all restaurants) and changes in BMI; the point estimate was a 0.026-unit increase in BMI (95% CI, −0.01 to 0.06) associated with a $1 increase in advertising per capita. However, when stratified by income level, we found a significant association for patients receiving health care services in low-income counties such that a $1 increase in restaurant advertising per capita was associated with a 0.053-unit increase in BMI (95% CI, 0.001-0.100). This association for patients receiving care in low-income counties appeared to be driven by full service restaurants and fast casual restaurants and by TV marketing because we observed significant associations with BMI for restaurant advertising among all media types for full service restaurants and fast casual restaurants (point estimate: 0.145; 95% CI, 0.06-0.23), advertising for all restaurants on TV only (point estimate: 0.057; 95% CI, 0.0002-0.11), and for fast casual restaurants and full service restaurants on TV only (point estimate: 0.141; 95% CI, 0.05-0.23). No significant associations were observed between restaurant advertising and BMI among patients receiving care in high-income counties. These results did not meaningfully change when we excluded patients assigned to rural counties (eTable 3 in the Supplement). The results from models distinguishing between the 6 media types (across all restaurants) and between the 3 restaurant types (across all media types) are included in eTable 4 in the Supplement.

**Table 2.** Association Between Restaurant Advertising and BMI, by Income, Restaurant Type, and Media Type^a^

Restaurant advertising	Overall (n = 29 285 920)			Low-income (n = 10 015 358)				High-income (n = 19 270 562)			
	Mean RA (mean change in RA) [10th-90th percentile]	β (95% CI)	*P* value	Mean RA (mean change in RA) [10th-90th percentile]	β (95% CI)	*P* value	Mean RA (mean change in RA) [10th-90th percentile]		β (95% CI)	*P* value
All media, all restaurants	4.73 (−0.16) [−0.58 to 0.27]	0.026 (−0.01 to 0.06)	.13	5.23 (−0.05) [−0.66 to 0.69]	0.053 (0.001 to 0.10)	.048	4.47 (−0.22) [−0.56 to 0.12]		0.017 (−0.02 to 0.05)	.36
All media by restaurant type										
Fast food	3.43 (−0.02) [−0.35 to 0.30]	−0.002 (−0.05 to 0.05)	.94	3.80 (0.06) [−0.38 to 0.53]	0.025 (−0.03 to 0.08)	.33	3.24 (−0.06) [−0.35 to 0.20]		−0.010 (−0.07 to 0.05)	.75
Fast casual and full service	1.30 (−0.33) [−0.59 to −0.03]	0.122 (0.04 to 0.21)	.005	1.42 (−0.33) [−0.69 to 0.04]	0.145 (0.06 to 0.23)	.001	1.23 (−0.33) [−0.56 to −0.05]		0.105 (−0.02 to 0.22)	.11
All restaurants by media type										
Television	4.17 (−0.31) [−0.82 to 0.11]	0.032 (−0.001 to 0.06)	.06	4.76 (−0.20) [−0.82 to 0.47]	0.057 (0.0002 to 0.11)	.049	3.68 (−0.37) [−0.82 to −0.04]		0.025 (−0.01 to 0.06)	.16
All other media	0.56 (−0.03) [−0.14 to 0.11]	−0.021 (−0.18 to 0.14)	.79	0.46 (−0.07) [−0.20 to 0.05]	0.058 (−0.09 to 0.21)	.45	0.61 (−0.02) [−0.11 to 0.11]		−0.058 (−0.25 to 0.13)	.54
By restaurant type and by media type										
TV										
Fast food	3.03 (0.03) [−0.30 to 0.34]	0.006 (−0.03 to 0.05)	.75	3.46 (0.13) [−0.24 to 0.57]	0.028 (−0.03 to 0.08)	.33	2.80 (−0.03) [−0.31 to 0.20]		0.007 (−0.04 to 0.05)	.76
Fast casual and full service	1.14 (−0.34) [−0.60 to −0.07]	0.114 (0.03 to 0.19)	.005	1.31 (−0.33) [−0.69 to 0.03]	0.141 (0.05 to 0.23)	.002	1.06 (−0.35) [−0.56 to −0.12]		0.090 (−0.03 to 0.21)	.13
All other media										
Fast food	0.41 (−0.05) [−0.14 to 0.06]	−0.139 (−0.48 to 0.20)	.42	0.34 (−0.07) [−0.19 to 0.01]	0.034 (−0.11 to 0.18)	.64	0.44 (−0.04) [−0.13 to 0.09]		−0.227 (−0.66 to 0.21)	.31
Fast casual and full service	0.15 (0.02) [−0.04 to 0.08]	0.212 (−0.06 to 0.48)	.12	0.12 (0.01) [−0.04 to 0.06]	0.179 (−0.30 to 0.66)	.47	0.17 (0.02) [−0.03 to 0.08]		0.211 (−0.09 to 0.51)	.17

Abbreviations: BMI, body mass index calculated as weight in kilograms divided by height in meters squared; RA, restaurant advertising.

^a^ Analysis of 29 285 920 person-quarters across 370 counties within athenahealth from 2013 to 2016 at the person-quarter level. The β coefficients, 95% CIs, and *P* values are the estimates for the association between restaurant exposure and BMI. The estimated associations each come from separate ordinary least-squares regression models with county fixed effects, controlling for median county income, educational level, and unemployment; individual race/ethnicity, sex, age group, and insurance type; with robust standard errors accounting for clustering at the county level.

**Figure 2.**
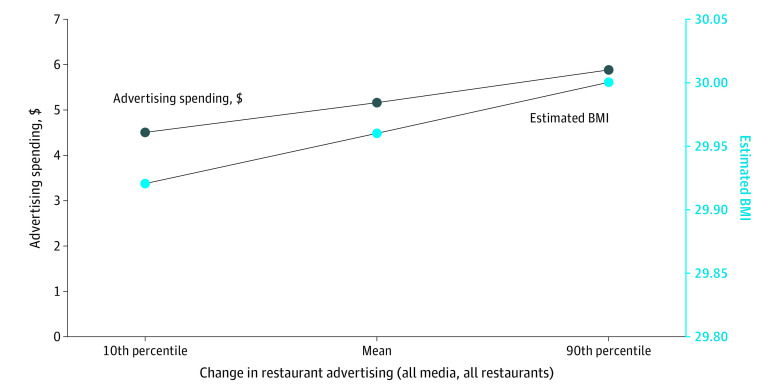
Changes in Body Mass Index (BMI) Associated With Relatively Large Decreases and Increases in Restaurant Advertising for Patients Who Received Health Care Services in Low-Income Counties From 2013 to 2016 Data are from patients included in athenahealth database and are analyzed at the person-quarter level (29 285 920 person-quarters). Three specific estimated values in BMI (calculated as weight in kilograms divided by height in meters squared) for 2016 (on the right-side axis) based on the regression result shown in the Table 2 models for adults receiving health care services in low-income counties. (This model is an ordinary least-squares regression for BMI as a function of quarterly advertising spending.) The first point shows the estimated BMI value corresponding to the 10th percentile in the change in estimated advertising from 2013 to 2016; the second, the estimated BMI value corresponding to the mean change in restaurant advertising; and the third, the estimated BMI value corresponding to the 90th percentile in the change in restaurant advertising. The left-side axis shows the corresponding amounts of restaurant advertising for 2016.

Figure 2 illustrates the magnitude of the observed association between restaurant advertising and BMI for those who received health care in low-income counties. In 2013, the mean quarterly restaurant advertising per capita was $5.17, and the mean BMI was 29.96 in low-income counties. Counties at the 10th percentile of the change in restaurant advertising per capita from 2013 to 2016 would have instead had restaurant advertising per capita of $4.52 in 2016; our model estimated a BMI equal to 29.92, or a decrease of 0.12%. Counties at the 90th percentile of the change in restaurant advertising per capita from 2013 to 2016 would have had restaurant advertising per capita of $5.87 in 2016; our model estimated a BMI equal to 30.00, or an increase of 0.12%. This difference in BMI between the counties facing relatively large decreases vs relatively large increases in exposure to restaurant advertising per capita corresponded to a total difference of 0.18 kg (0.41 pounds) per person.

## Discussion

This is the first study, to our knowledge, to use national data with objectively measured BMI to examine whether changes in restaurant advertising expenditures over time were associated with changes in BMI. We found that restaurant advertising spending was positively associated with weight gain for patients in low-income counties, but not in high-income counties. The magnitude of the observed association (ie, an increase of 0.18 kg [0.41 pounds] between those facing a change in restaurant advertising at the 90th percentile instead of the 10th percentile) was small but seemed reasonable given our study design’s use of the variation in changes in exposure over time. It is plausible that the focus on population means masks important variation among individuals, or misclassification of the primary estimator may bias our results toward the null. Such misclassification would have to be time-varying and differential within higher- and lower-income counties.

The positive association between restaurant advertising and weight gain for patients in low-income counties (but not in high-income counties) could potentially be explained by the types of foods and beverages marketed to this group being relatively less healthy than those marketed to patients in high-income counties.^6^ This explanation is consistent with prior evidence indicating that advertisements for unhealthy items are often targeted toward those at higher risk for obesity,^2,3^ and unhealthy food retail outlets, such as fast food restaurants, are more densely located in low-income and minority communities.^33^ Although our analysis did not examine differences in advertising spending by the nutrient composition of menu items, prior research suggests that exposure to food-related advertisements, particularly for sugary beverages and fast food restaurants, is significantly higher among youth in communities with higher proportions of Black families and lower-income families.^6^ Moreover, TV advertising accounts for the majority of advertising spending per capita, and prior research found that targeted TV marketing of food and beverages toward Black children or Hispanic children were for products high in fat or sugar.^34^

The findings of this study are concerning because they suggest that restaurant advertising spending targeted toward low-income communities may contribute to the observed disparities in obesity.^8^ Given that the vast majority of the research examining the association between marketing and obesity has focused on children,^1,2,15,16,17,18,19,20,21,22^ more research focused on adults would be helpful (eg, nutrition content of menu items being advertised toward adults in low-income communities).

Examined alongside previous research showing that unhealthy food and beverage marketing is associated with higher obesity risk in youth,^1,2,15,16,17,18,19,20,21,22^ our findings suggest that public policies or initiatives or private sector actions would help reduce the negative impacts of exposure to restaurant advertising across the population. These results specifically point to the potential benefits from reduced exposure to restaurant advertising among adults with low incomes.

### Limitations

Our study has limitations. First, our restaurant sample was limited to 66 of the 100 largest US chain restaurants and may not generalize to other restaurant types (eg, small chain, locally owned, or fine dining restaurants) which may spend relatively less on advertising. Second, restaurant advertising expenditures of less than $50 in a DMA were not included in the Kantar Media data, likely generating noise in our estimates of total restaurant exposure. Third, our measure of advertising per capita did not take into account the relative popularity of items (because sales data were not incorporated), the type of advertisement promotion (eg, pricing deal or new menu item) or the content of the advertising (eg, types of graphical displays). Moreover, disaggregating the national and DMA-level measures of Kantar Media advertising into county-level measures incorporating specific restaurant locations may introduce complications in interpreting the results, although this concern should be relatively limited in the present longitudinal analyses in which the variation was largely driven by changes in quarterly advertising over time. Fourth, whereas the sample of participants included in this analysis represented a large number of individuals followed up for more than 4 years, the sample was relatively homogeneous, with a small representation of non-White individuals (12% of patient-quarters) and adults younger than 40 years (30% of patient-quarters), and only represented individuals who already had some level engagement with the health care system. Despite our data coming from 44 states (including 8 of the 10 most populous US cities in 2016), we had patient data only from 370 urban/suburban counties, which also reduces generalizability. Fifth, although we accounted for individual random effects and county-level controls, our models may still have an omitted variable bias from not being able to control for confounders such as dietary intake and physical activity. Therefore, it is possible that the observed associations were driven by restaurant chains increasing advertising in the neighborhoods with the greatest number of at-risk individuals. Sixth, county of residence, income, and educational level were unavailable for patients in the athenahealth data. Therefore, counties were assigned based on where medical services were rendered, and income and educational level were assigned based on the patient’s county. The major strengths of this study included the use of unique local restaurant advertising data, including multiple types (eg, TV, radio, and online), combined with a longitudinal design and our use of objectively measured BMI data, which do not have the risk of self-report bias in which individuals often underreport their weight.^35^

## Conclusions

In conclusion, the present study found that restaurant advertising was associated with modest weight gain among adults in low-income counties—communities which are often exposed to disproportionately more advertising for unhealthy menu items and which are composed of individuals at higher risk for obesity. Efforts to decrease the promotion of unhealthy menu items targeting low-income communities should be intensified to help attenuate the association of restaurant advertising with obesity risk among vulnerable populations.

## Notes

Supplement. **eTable 1.** Description of Measures and Data Sources for the Analysis **eFigure.** athenahealth Sample Selection **eAppendix.** Calculating the Per Capita County-Level Restaurant Advertising Expenditure **eTable 2.** Total Restaurant Advertising Expenditure for Parker County, TX, Quarter 1, 2013 **eTable 3.** Association Between Restaurant Advertising (RA) and BMI, by Income, Restaurant Type, and Media Type for Urban-Only Sample **eTable 4.** Association Between Restaurant Advertising (RA) and BMI, by Income, Detailed Restaurant Type, and Detailed Media Type
