# Supplementary material for: Association of Chain Restaurant Advertising Spending With Obesity in US Adults
Source: JAMA Netw Open. 2020 Oct 7;3(10):e2019519. doi: 10.1001/jamanetworkopen.2020.19519 (PMC7542328; doi:10.1001/jamanetworkopen.2020.19519)
Supplement: Supplement. — eTable 1. Description of Measures and Data Sources for the Analysis eFigure. athenahealth Sample Selection eAppendix. Calculating the Per Capita County-Level Restaurant Advertising Expenditure eTable 2. Total Restaurant Advertising Expenditure for Parker County, TX, Quarter 1, 2013 eTable 3. Association Between Restaurant Advertising (RA) and BMI, by Income, Restaurant Type, and Media Type for Urban-Only Sample eTable 4. Association Between Restaurant Advertising (RA) and BMI, by Income, Detailed Restaurant Type, and Detailed Media Type [file jamanetwopen-e2019519-s001.pdf]

Supplementary Online Content

Bleich SN, Soto MJ, Jones-Smith JC, et al. Association of chain restaurant advertising spending with obesity in US adults. *JAMA Netw Open*. 2020;3(10):e2019519. doi:10.1001/jamanetworkopen.2020.19519

eTable 1. Description of Measures and Data Sources for the Analysis

eFigure. athenahealth Sample Selection

eAppendix. Calculating the Per Capita County-Level Restaurant Advertising Expenditure

eTable 2. Total Restaurant Advertising Expenditure for Parker County, TX, Quarter 1, 2013

eTable 3. Association Between Restaurant Advertising (RA) and BMI, by Income, Restaurant Type, and Media Type for Urban-Only Sample

eTable 4. Association Between Restaurant Advertising (RA) and BMI, by Income, Detailed Restaurant Type, and Detailed Media Type

This supplementary material has been provided by the authors to give readers additional information about their work.

eTable 1. Description of Measures and Data Sources for the Analysis

Indicator	Unit	Description	Source
**Outcome**			
Body mass index	Individual-level	Measured BMI	athenahealth, 2013-2016
**Independent variable of interest:** Exposure to restaurant advertising			
Restaurant advertising	Restaurant-level	Total quarterly advertising spend	Kantar Media, 2013-2016 (restaurant name obtained from MenuStat)
Restaurant locations	Restaurant-level	Zip code of each restaurant location	AggData, 2013-2016 (restaurant name obtained from MenuStat)
**Covariates**			
Demographics	Individual-level	Age, gender, race, insurance type	athenahealth, 2013-2016
Population characteristics	County-level	County population	2013-2016 U.S. Census Bureau's American Community Survey

NOTES: Athenahealth is a health care technology and services company connecting more than 85,000 medical providers from mostly outpatient care settings. De-identified patient data were obtained from athenahealth's ambulatory electronic health record in which a unique randomized identifier is used to track patients over time. Participant data included the county where services were rendered, race/ethnicity, gender, age, BMI, and date of each visit. Kantar Media is a data and consulting company that provides analysis of print, radio, TV, outdoor and social media advertising expenditures. AggData (www.aggdata.com) is a business locational dataset which provides the zip code locations for these 66 different restaurant chains in each year. U.S. Census Bureau's American Community Survey is a publicly-available dataset which provides detailed population information about each county.

© 2020 Bleich SN et al. *JAMA Network Open*.

eFigure: athenahealth Sample Selection

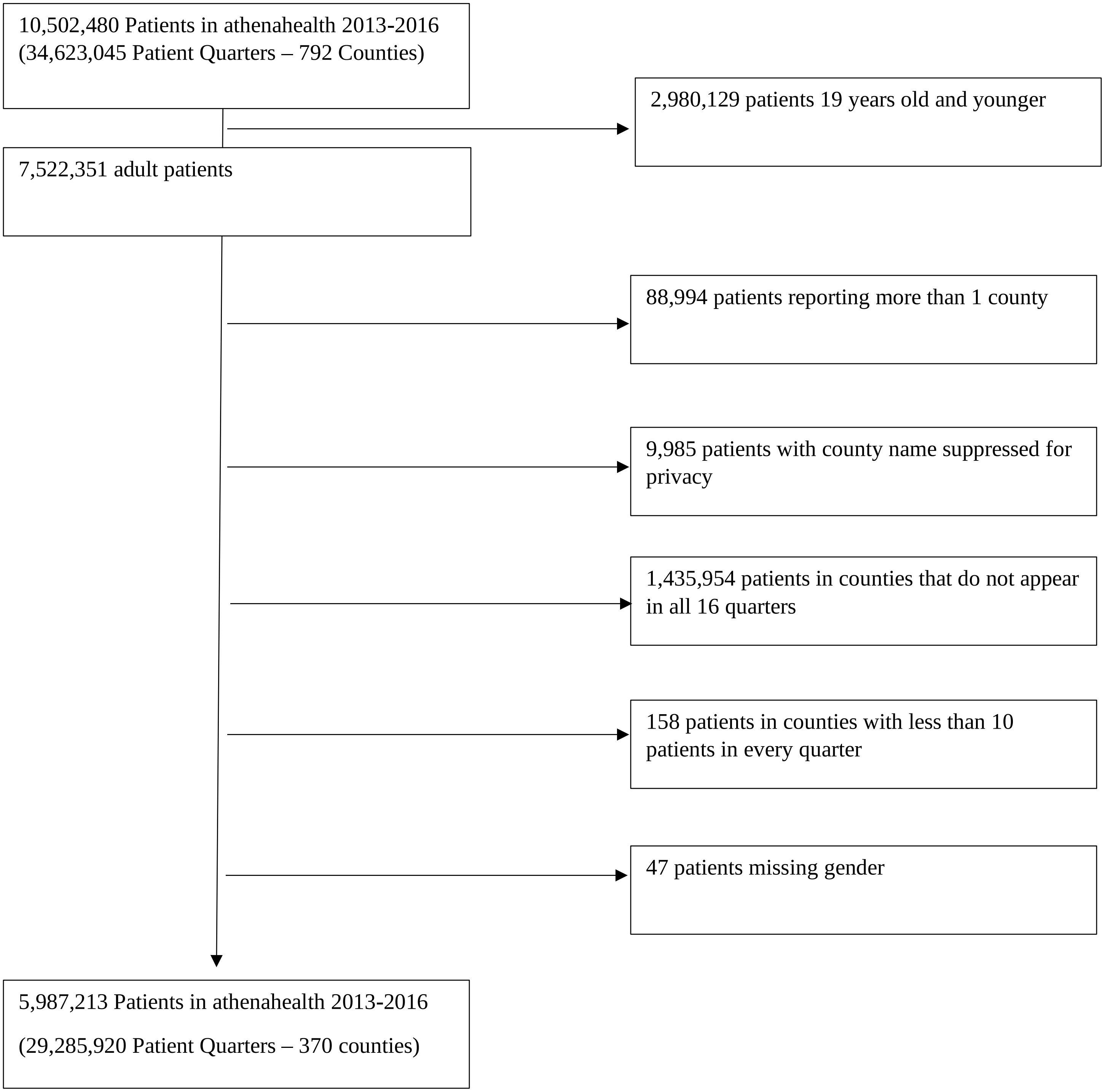

© 2020 Bleich SN et al. *JAMA Network Open*.

eAppendix. Calculating the Per Capita County-Level Restaurant Advertising Expenditure

Designated Market Areas, developed by Nielsen Media Research, divide the United States into distinct groups of counties, which generally surround urban areas and receive the same broadcast television. Of note, counties do not always lie exclusively within a single DMA; nationwide, seven counties are split between DMAs. A subset of adult patients included in our sample received healthcare in two of those seven counties: Kern County, CA & Riverside County, CA. For the purpose of this analysis, counties were each assigned to a single DMA [Kern to the Bakersfield DMA, and Riverside to the Los Angeles DMA] based on proportion of county land within each of the two DMAs represented by the county. Kantar Media provides quarterly advertising expenditure for restaurants in the 210 individual Designated Market Areas (DMA) in addition to nationwide advertising expenditure. For a given restaurant *i*, nationwide advertising is divided evenly among all DMAs in which that restaurant is located. Per capita advertising expenditure for county *c* (within DMA *d*) and quarter *q* (within year *y*) is calculated with the following equation:

$$Per\ capita\ restaurant\ advertising_{cq} = \frac{\sum_{i=1}^{N}\left(\frac{Restaurant\ locations_{icy}}{Restaurant\ locations_{idy}} * Total\ DMA\ expenditure_{idq}\right)}{county\ population_{cy}}$$

where the proportion of locations for restaurant chain *i* (e.g., McDonald's) within county *c* in year *y* of all locations for restaurant *i* within DMA *d* in year *y* is multiplied by the total advertising expenditure by restaurant *i* within DMA *d* in quarter *q* of year *y*. The proportional expenditure for each restaurant *i* is then summed across all restaurant chains, *N*, (recognizing that some chains simply have no locations and/or adverting within any given county) and then divided by the total population in county *c* in year *y*.

Restaurant advertising expenditures are also be categorized by media type (TV, internet, radio, print, outdoor and business-to-business) and by restaurant type (fast food, fast casual, full-service).

Per capita restaurant advertising expenditure calculation example

To illustrate how the restaurant advertising measure is calculated, consider Parker County in Texas; it was the county with the median per capita restaurant advertising expenditure in quarter 1 of 2013. It belongs to the Dallas DMA. Appendix Table C1 shows each restaurant chain in Parker County in 2013, along with that restaurant's total DMA advertising expenditure, its number of store locations in the county in 2013, and the proportional advertising expenditure stratified by TV and all other media types. As indicated at the bottom of this table, the sum of the proportional advertising equals $591,974 for quarter 1 of 2013. With a total population of 121,418 in 2013, the resulting per capita restaurant advertising expenditure is:

$$Per\ capita\ restaurant\ advertising\ expenditure_{\text{Parker,2013Q1}} = \frac{\$591{,}974}{121{,}418} = \$4.88$$

© 2020 Bleich SN et al. *JAMA Network Open*.

eTable 2. Total Restaurant Advertising Expenditure for Parker County, TX, Quarter 1, 2013

Restaurant	Total DMA Spending	Number of Locations in County	TV Media	All Other media	Total
**Fast Food**	**26,830,980**	**41**	**350,591**	**62,567**	**413,158**
Arbys	363,821	1	3,222	2,944	6,166
Burger King	1,229,396	1	10,151	2,143	12,294
Chik-fil-A	307,092	1	1,456	2,156	3,613
Dairy Queen	479,892	3	13,120	2,195	15,316
Domino’s	1,454,291	2	22,917	730	23,647
Jack in the Box	1,466,613	2	10,400	5,038	15,438
Little Caesers	926,757	1	8,081	622	8,743
Long John Silver’s	386,828	1	8,792	-	8,792
McDonalds	4,872,256	4	47,495	15,170	62,666
Papa John’s	836,226	1	12,813	896	13,709
Sonic	4,691,634	6	93,122	90	93,211
Subway	4,235,947	12	63,120	19,533	82,653
Taco Bell	2,766,235	2	29,977	1,458	31,434
Wendy’s	1,569,193	1	8,674	2,865	11,538
Whataburger	1,244,800	3	17,252	6,687	23,938
**Fast Casual**	**1,223,009**	**3**	**9,376**	**2,188**	**11,563**
Cici’s	577,123	1	7,214	-	7,214
Starbucks	645,886	2	2,162	2,188	4,349
**Full Service**	**7,631,590**	**8**	**157,776**	**9,477**	**167,253**
Applebee’s	1,106,253	1	25,018	2,638	27,656
Chili’s	1,949,651	1	27,390	70	27,460
Denny’s	466,129	1	9,876	258	10,133
IHOP	565,831	1	8,141	1,615	10,133
Olive Garden	1,512,908	1	56,610	3,906	60,516
Pizza Hut	2,030,818	3	30,741	991	31,372
**Total**	**35,685,579**	**52**	**517,742**	**74,232**	**591,974**

© 2020 Bleich SN et al. *JAMA Network Open*.

eTable 3. Association Between Restaurant Advertising (RA) and BMI, by Income, Restaurant Type, and Media Type for Urban-Only Sample

	Overall N=28,615,414			Low-income N=11,009,750			High-income N=17,605,704		
**Restaurant Advertising**	**Mean RA (Mean ΔRA) [10th : 90th]**	**Estimate (95% C.I.)**	**p-values**	**Mean RA (Mean ΔRA) [10th : 90th]**	**Estimate (95% C.I.)**	**p-values**	**Mean RA (Mean ΔRA) [10th : 90th]**	**Estimate (95% C.I.)**	**p-values**
All Media, All Restaurants	4.74 (-0.18) [-0.59 : 0.23]	0.030 (-.01, .06)	.10	5.28 (-0.09) [-0.67 : 0.56]	0.069 (.02, .12)	.009	4.40 (-0.23) [-0.82 : 0.08]	0.004 (-.03, .04)	.85
**All Media by Restaurant Type**									
All Media, Fast Food	3.43 (-0.03) [-0.36 : 0.29]	-0.002 (-.05, .05)	.95	3.80 (0.05) [-0.35 : 0.48]	0.038 (-.01,.09)	.15	3.19 (-0.07) [-0.36 : 0.20]	-0.029 (-.09, .04)	.39
All Media, Fast Casual + Full Service	1.31 (-0.33) [-0.60 : -0.04]	0.136 (.04, .23)	.004	1.44 (-0.32) [-0.62 : 0.00]	0.179 (.08, .28)	<.001	1.19 (-0.32) [-0.56 : -0.09]	0.09 (-.03, .22)	.15
**All Restaurants by Media Type**									
TV, All Restaurants	4.18 (-0.32) [-0.79 : 0.09]	0.036 (0.001, .07)	.04	4.80 (-0.23) [-0.76 : 0.46]	0.075 (.02, .13)	.01	3.78 (-0.39) [-0.79 : -0.08]	0.010 (-.02, .05)	.46
All other media, All Restaurants	0.57 (-0.03) [-0.14 : 0.10]	-0.018 (-0.19, 0.15)	.84	0.48 (-.07) [-0.19 : 0.04]	0.062 (-.10, .22)	.45	0.62 (-0.01) [-0.11 : 0.14]	-0.088 (-.31, .13)	.43
**By Restaurant Type and by Media Type**									
TV, Fast Food	3.02 (0.02) [-0.27 : 0.32]	0.010 (-.03, .05)	.74	3.44 (0.12) [-0.20 : 0.54]	0.042 (-.01, .10)	.14	2.75 (-0.04) [-0.27 : 0.18]	-0.008 (-.06, .04)	.76
TV, Fast Casual + Full Service	1.16 (-0.35) [-0.60 : -0.09]	0.128 (.04, .21)	.004	1.36 (-0.35) [-0.70 : 0.00]	0.176 (.08, .28)	.001	1.04 (-0.35) [-0.55 : -0.14]	0.080 (-.04, .20)	.17
All other media, Fast Food	0.41 (-0.05) [-0.14 : 0.05]	-0.138 (-.50, .23)	.46	0.36 (-0.07) [-0.16 : 0.01]	0.040 (-.11, .20)	.61	0.45 (-0.04) [-0.13 : 0.09]	-0.271 (-.78, .24)	.29
All other media, Fast Casual + Full Service	0.16 (0.02) [-0.04 : 0.08]	0.217 (-.05, .49)	.11	0.13 (0.003) [-0.04 : 0.06]	0.176 (-.28, .63)	.45	0.17 (0.02) [-0.03 : 0.09]	0.196 (-.11, .50)	.20

SOURCE: Authors' analysis of athenahealth patients from 2013-2016 at the person-quarter level (N=28,615,414 across 331 counties).

NOTES: The beta coefficients, confidence internals, and p-values are the estimates for the association between restaurant exposure and BMI. The estimated associations each come from separate OLS regression models with county fixed effects, controlling for median county income, education, and unemployment; individual race, sex, age group and insurance type; with robust standard errors accounting for clustering at the county level.

© 2020 Bleich SN, et al. *JAMA Network Open*.

eTable 4. Association Between Restaurant Advertising (RA) and BMI, by Income, Detailed Restaurant Type, and Detailed Media Type

	Overall N=29,285,920			Low-income N=10,015,358			High-income N=19,270,562		
RA Measure	Mean RA (Mean ΔRA) [10th : 90th]	Estimate (95% C.I.)	p-value	Mean RA (Mean ΔRA) [10th : 90th]	Estimate (95% C.I.)	p-value	Mean RA (Mean ΔRA) [10th : 90th]	Estimate (95% C.I.)	p-value
**All Restaurants by Media Type**									
TV, All Restaurants	4.17 (-0.31) [-0.82 : 0.11]	0.036 (-0.001, .06)	.06	4.76 (-0.20) [-0.82 : 0.47]	0.057 (.0002, .11)	.05[1]	3.68 (-0.37) [-0.82 : -0.04]	0.025 (-.01, .06)	.16
Internet, All Restaurants	0.08 (-0.05) [-0.08 : -0.03]	0.093 (-.40, .58)	.71	0.08 (-0.05) [-0.08 : -0.03]	0.394 (-.29, 1.1)	.25	0.08 (-0.05) [-0.07 : 0.12]	0.080 (-.57, .73)	.81
B2B, All Restaurants	0.001 (-0.0003) [-0.001 : 0.0002]	-7.62 (-23, 8.8)	.38	0.001 (-0.0003) [-0.001 : 0.0002]	-17.98 (-36, .11)	.05	0.001 (-0.0003) [-0.001 : 0.0002]	15.01 (-12, 42)	.28
Outdoor overall, All Restaurants	0.16 (0.01) [-005 : 0.08]	0.179 (.001, .36)	.05[1]	0.16 (-0.01) [-0.06 : 0.07]	0.159 (-.02, .45)	.07	0.16 (0.02) [-0.04 : 0.08]	0.201 (-.06, .46)	.13
Print overall, All Restaurants	0.10 (-0.02) [-0.05 : 0.01]	-0.104 (-.53, .32)	.63	0.09 (-0.03) [-0.06 : -0.004]	-0.025 (-.31, .27)	.86	0.10 (-0.02) [-0.05 : 0.01]	-0.050 (-.56, .46)	.51
Radio overall, All Restaurants	0.22 (0.03) [-0.03 : 0.12]	-0.19 (-.49, .11)	.21	0.13 (0.02) [-0.04 : 0.06]	-0.106 (-.51, .30)	.61	0.27 (0.03) [-0.03 : 0.13]	-0.281 (-.62, .06)	.11
**All Media by Restaurant Type**									
All Media, Fast Food	3.43 (-0.02) [-0.35 : 0.30]	-0.002 (-.05, .05)	.94	3.80 (0.06) [-0.35 : 0.53]	0.025 (-.03, .08)	.33	3.24 (-0.06) [-0.35 : 0.20]	-0.010 (-.07, .05)	.75
All Media, Fast Casual	0.26 (0.08) [0.09 : 0.48]	-0.016 (-.19, .15)	.85	0.22 (0.12) [0.01 : 0.24]	0.121 (-.06, .30)	.19	0.28 (0.07) [-0.01 : 0.15]	-0.005 (-.18, .17)	.95
All Media, Full Service	1.05 (-0.41) [-0.70 : -0.13]	0.163 (.03, .29)	.01	1.21 (-0.44) [-0.78 : -0.05]	0.167 (.07, .26)	.001	0.95 (-0.39) [-0.61 : -0.15]	0.133 (-.05, .32)	.15

SOURCE: Authors' analysis of athenahealth patients from 2013-2016 at the person-quarter level (N=29,285,920 across 370 counties).

NOTES: The beta coefficients, confidence internals, and p-values are the estimates for the association between restaurant exposure and BMI. The estimated associations each come from separate OLS regression models with county fixed effects, controlling for median county income, education, and unemployment; individual race, sex, age group and insurance type; with robust standard errors accounting for clustering at the county level.

[1] This p-value is statistically significant at $p < 0.05$ when carried out to three decimal points.

© 2020 Bleich SN, et al. *JAMA Network Open*.
